# Palliative Care for a Mentally Incompetent End Stage Renal Failure Patient: Why Is It Important?

**DOI:** 10.1155/2015/478783

**Published:** 2015-03-09

**Authors:** Kwok-Ying Chan, Terence Yip, Mau-Kwong Sham, Benjamin Hon-Wai Cheng, Cho-Wing Li, Yim-Chi Wong, Vikki Wai-Kee Lau

**Affiliations:** ^1^Palliative Medical Unit, Grantham Hospital, Wong Chuk Hang, Hong Kong; ^2^Renal Unit, Department of Medicine, Tung Wah Hospital, Sheung Wan, Hong Kong

## Abstract

People with intellectual disabilities are among the most disadvantaged groups in society. Here we report a mentally incompetent end stage renal failure (ESRF) patient with frequent emergency visits who made a significant improvement in symptoms control and reduction in casualty visits after introduction of renal palliative care service. Multidisciplinary approach would be useful in this case.

## 1. Introduction

As the risk of life-limiting diseases such as end stage renal disease (ESRD), it could be speculated that more people with intellectual disabilities now suffer such disease and may require palliative care if they are chosen for conservative therapy without dialysis [1]. Here we report a mentally incompetent ESRD patient with frequent emergency visits who made a significant improvement in symptoms control and reduction in casualty visits after introduction of renal palliative care service.

## 2. Case Report

Mr. K was a 53-year-old gentleman with history of diabetes mellitus and hypertension since his teenage years. His diabetic control had never been satisfactory and he suffered from end stage kidney disease since his 50s.

He was mildly mental incapacitated and received special education since 3 years old. Besides, his mother had moderate to severe dementia who requires regular medication and constant attention. His father died when he was young and his elder sister suffered from Parkinsonism at her 40s. Since his mother was a landlady, there was no financial difficulty for this family. He received formal assessment by nephrology team but was found to be difficult in hemodialysis (HD) due to the patient being unable to keep still during therapy. In addition, due to his complicated social history and lack of support, peritoneal dialysis (PD) could not be managed by the family. After a thorough discussion with his family, it was decided that his ESRF should be managed conservatively without dialysis.

His drug and fluid compliance was all along poor. Besides, he had attention seeking behavior related to his mental incompetence. Prior to the introduction of renal palliative care, he had frequent emergency department (ED) visits due to various reasons, including gouty attack (plasma urate = 6.5 mg/dL), dizziness, edema, pruritus, fatigue, and severe hyperkalemia (*K* = 7.3 mmol/L). Some of the episodes were of minor complaints and he could be discharged home without requiring ward admission. Family members and domestic helper mentioned that this had caused significant stress to them.

His baseline laboratory results revealed the following: hemoglobin level (Hb) 8.1 g/dL; plasma creatinine level 9.5 mg/dL; phosphate 5.8 mg/dL, calcium 8.5 mg/dL; PTH 23 pmol/L; and eGFR 9 mL/min/1.73 m^2^. His blood gas showed pH 7.39, HCO3^−^ 26.1 mmol/L and base excess 1 mmol/L.

After ruling out vitamins and iron deficiency, continuous erythropoietin receptor activator (CERA) which was a long acting erythropoietin stimulating agent (ESA) was started in the palliative care clinic and titrated against his body weight and Hb level [2]. His uremic pruritus was treated with sertraline which was then escalated to 150 mg according to clinical response [3]. His limb edema was reduced after using a combination of diuretic and reinforcement of drug and fluid compliance [4]. Gouty attack was treated with analgesics and ice pack. Diet advice and allopurinol were given to prevent further attack.

Home care nurse was referred for psychological interventions while social worker was referred for social support to the patient and his family members. In addition, he was arranged to join the activities in our palliative care day centre. The patient and his domestic helper highly appreciated our arrangement. In the day centre, Mr. K could have social gathering with other patients, for example, enjoying opera and music video, watching television, doing some hand-made work, and joining outdoor activities, for example, going to Disneyland. In fact, he has high attendance rates (about 2-3 times/week) in our centre.

After 6 months of care, the severity of pain, edema, and pruritus were significantly reduced (5 to 2, 7 to 3, and 8 to 2 out of 10, resp.) according to Edmonton Symptom Assessment Scale (ESAS) [5]. Hb level was raised to 10-11 mg/dL. His 6-month casualty attendance was markedly reduced from 13 to only 1 time after receiving renal PC care because his uremic symptoms and psychosocial needs could now be better managed via our interdisciplinary approach.

## 3. Discussion

This report demonstrated how renal palliative care could help relieve symptom, provide psychosocial care, and thus reduce healthcare cost through multidisciplinary approach.

Mr. K suffered from heavy symptom burden which was commonly found in renal palliative care patients [6]. By using ESA, it was shown to reduce fatigue for this group of patients by correcting anaemia [2]. Sertraline, a selective serotonin reuptake inhibitor, was found to be effective to relieve antihistamine refractory uremic pruritus and help reduce anxiety when the dosage is titrated to above 100 mg [3]. For treatment of refractory fluid retention, addition of low dose metolazone to furosemide could be successfully managed in the previous studies [4]. In order to prevent episodes of hyperkalemia and fluid overload, diet and drug compliance were both reinforced by doctor and our nurses visit during his PC clinic follow-up. By using collaborative care model between nephrology and palliative care team, symptom scores, depressed mood, and the rate of rehospitalization were found to be reduced in our local study [7].

A previous study found that many chronically ill people who were admitted to an ED had complex psychosocial problems and limited access to support systems and homecare services along with their medical problems [8]. PC services possess superiority over EDs and acute hospital setting for a holistic, multidisciplinary, and timely approach for addressing PC needs of renal failure patients. The role of day hospice care to renal palliative care patients has never been mentioned previously. Apart from providing social support and respite care, regular monitoring of patient's diet and fluid compliance by our staff of day centre might alleviate fluid overload or gouty attack for this patient.

In fact, people with intellectual disabilities are among the most disadvantaged groups in society. Mild intellectual disabilities are particularly vulnerable as their intellectual disability is not always recognized [9]. They may be socially clumsy and misread during social situations, which could lead healthcare staff to adopt negative attitudes.

Pertinent issues included the fact that their somatic complaints were often thought to be related to their mental incapacitation and hence ignored. Difficulty for the family in finding medical teams which could cope with Mr. K's dual needs is as well demonstrated in this case report. A previous study had shown that medical assessment for this group of patients is difficult due to communication problems, lack of medical notes and history due to previously fragmented care, and unfamiliarity of staff in ordinary healthcare services in dealing with people with intellectual disabilities [1].

Carers who have worked closely with the person with intellectual disabilities may also have strong feelings and emotions of their own when their client becomes terminally ill, complicating the situation further [1]. Botsford suggested that the complexity of staff roles, where there is a responsibility not only to individuals and families but also to colleagues, administrations, regulating bodies, and other providers, creates conflicts and dilemmas about end-of-life care [10]. If there is a disagreement between different parties, she suggested the involvement of social workers, clergy, hospice workers, or bereavement professionals to work as mediators. Palliative care professionals can be in a good position to provide guidance and support in such situations.

It is foreseeable that as patients become frailer, multidisciplinary input will play a central role in addressing their needs, including symptom control, advance care planning, and end-of-life care. It is crucial that palliative care be well coordinated to address the needs of these patients. A similar success by using this collaborative model can be seen with hematology teams of local hospitals [11]. Our joint care model in Hong Kong provided a good learning opportunity for both specialists in palliative care and nephrology, and, hopefully, patients will benefit from our joint effort in care.
